# Recombinant Influenza Vaccines 

**Published:** 2012

**Authors:** E.S. Sedova, D.N. Shcherbinin, A.I. Migunov, Iu.A. Smirnov, D.Iu. Logunov, M.M. Shmarov, L.M. Tsybalova, B.S. Naroditskiĭ, O.I. Kiselev, A.L. Gintsburg

**Affiliations:** Gamaleya Research Institute of Epidemiology and Microbiology, Gamaleya Str., 18, Moscow, Russia, 123098; Research Institute of Influenza, prof. Popov Str., 15/17, Saint Petersburg, Russia, 197376; Ivanovsky Research Institute of Virology, Gamaleya Str., 16, Moscow, Russia, 123098

**Keywords:** Recombinant vaccine, influenza, immunization

## Abstract

This review covers the problems encountered in the construction and production of new recombinant influenza vaccines. New approaches to the development of influenza vaccines are investigated; they include reverse genetics methods, production of virus-like particles, and DNA- and viral vector-based vaccines. Such approaches as the delivery of foreign genes by DNA- and viral vector-based vaccines can preserve the native structure of antigens. Adenoviral vectors are a promising gene-delivery platform for a variety of genetic vaccines. Adenoviruses can efficiently penetrate the human organism through mucosal epithelium, thus providing long-term antigen persistence and induction of the innate immune response. This review provides an overview of the practicability of the production of new recombinant influenza cross-protective vaccines on the basis of adenoviral vectors expressing hemagglutinin genes of different influenza strains.

## INTRODUCTION 

Influenza is the most common infectious disease. According to the WHO, 20–30% of children and 5–10% of adults are infected with influenza annually; the severe complications caused by it result in the death of 250, 000-500, 000 people. The economic burden inflicted by influenza epidemics is estimated at 1–6 million USD per 100, 000 population [1]. The burden and mortality increase significantly during pandemics. Thus, according to different sources, the influenza pandemic that struck in 1918–1919 caused 50-100 million deaths [2]. 

Prevention through vaccination is the most sensible measure to protect people against influenza and to contain its spread [3]. Modern influenza vaccines typically induce the formation of antibodies against the influenza virus’ surface antigens: hemagglutinin (HA) and neuraminidase (NA). These vaccines include both live and inactivated (whole-virion, split, subunit) vaccine types. The efficiency of seasonal vaccines directly depends on the degree of correspondence between the antigenic structure of the influenza virus strains within the vaccine and the strains circulating among the population during a given epidemic season. The influenza virus surface proteins undergo progressive antigenic variation (antigenic drift), thus requiring annual renewal of the strain composition of vaccines [4]. 

The development of highly immunogenic and safe vaccines inducing the immune response of a broad spectrum of action is currently one of the major problems encountered in efficient influenza prevention. The 2009–2010 pandemic caused by the influenza A(H1N1)pdm09 virus and the existing pandemic threat of avian influenza A(H5N1) sustain the interest in designing new vaccines capable of inducing broad protective immunity [5]. 

**The use of reverse genetics techniques to design influenza vaccines **

The existing influenza vaccines can be subdivided into two types: the attenuated (live) and inactivated (including subunit) types. All of those are rather widely used for population immunization and have shown themselves to perform well. Attenuated vaccines are influenza viruses with attenuated virulence [6]. The epidemically topical virus strains are also used to produce inactivated subunit vaccines, although the use of high pathogenic strains is limited by strict requirements imposed on the biological safety of the production process [7]. 

**Fig. 1:**
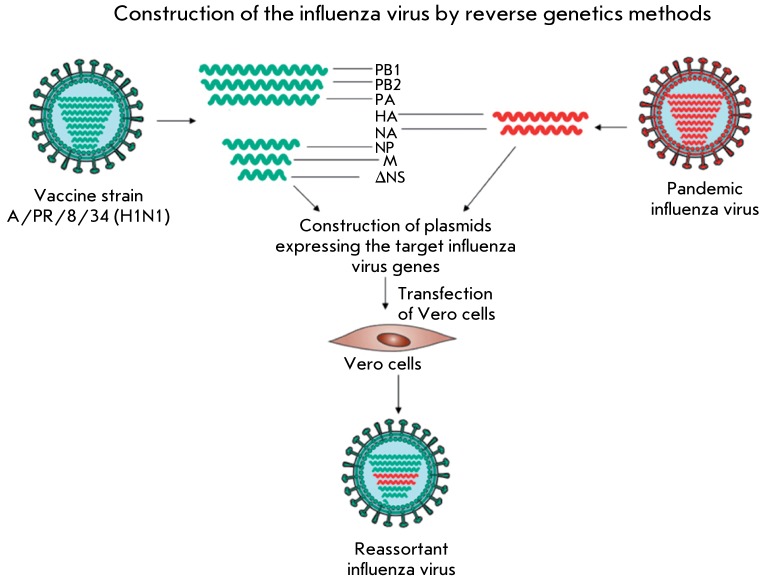
Production of a new influenza reassortant strain by reverse genetics methods

The conventional methods for producing vaccine strains of the influenza A virus have a number of drawbacks. The use of both attenuation based on viral adaptation to the organism of the heterologous host [8] and reassortment after coinfection with epidemic strains and attenuation donors [9] does not always make it possible to preserve the equilibrium between the virulence level of the original virus and its immunogenicity. Excessive attenuation may result in the production of strains that have lost all ability to reproduce in the cells of the human respiratory tract. 

The use of reverse genetics techniques is an alternative method for obtaining vaccine strains. Reverse genetics techniques allow to reconstruct a biologically active viral particle by coinfecting permissive cell lines with plasmids containing the genes that encode viral proteins. The virulence and antigenic properties of the influenza virus can be manipulated by altering these genes [10]. 

The reverse genetics techniques can be used to obtain reassortant influenza viruses. Thus, the plasmids encoding the segments in the genomes of pandemic or circulating seasonal strains and the attenuated vaccine strain of the influenza A virus are used to transfect permissive eukaryotic cells. As a result, the assembly of whole virions of the virus carrying a combination of proteins of both the vaccine and pathogenic strains occurs ( *Fig. 1* ). The influenza A(H1N1) virus that caused the 1918 pandemic (the so-called Spanish Influenza) was successfully obtained and examined using this very technique [11]. 

Reverse genetics techniques allow to reduce the viral virulence by introducing mutations into various viral genes. Thus, mutations in two genes encoding the polymerase proteins PB1 and PB2 of the avian influenza A/guinea fowl/Hong Kong/WF10199 (H9N2) virus caused the loss of viral pathogenicity for chickens [12]. The deletion of the nonstructural protein NS1 resulted in attenuation of the influenza A virus. The vaccine obtained using this method has successfully undergone phase I clinical trials [13, 14]. The introduction of mutations to the M2 protein, which is essential for ion channel formation, also results in virus attenuation [15]. After variation of the amino acid sequence at the fragmentation sites of HA of the highly pathologic influenza A H5 virus by targeted mutagenesis, the virus acquires the characteristics of low pathogenic viruses [16]. 

The reverse genetics techniques have shown good results in obtaining attenuated strains of the influenza virus [17]. However, the use of reassortment in the case of vaccine strains brings to the fore the question of biosafety because of possible mutations that can recover or increase the viral virulence [18]. Furthermore, the extensive use of live attenuated influenza vaccines casts suspicion because of the possible reassortment of a live vaccine with the circulating strains of human influenza viruses [19, 20]. Vaccine strains of the influenza virus in preparative amounts are most commonly produced in chicken embryos, which makes vaccination of individuals allergic to chicken protein impossible. Another drawback of the vaccines produced using chicken embryos is the dependence of the technological process on fertility in the chicken flock. 

**Recombinant subunit vaccines **

The problems associated with the use of chicken embryos and the necessity to attenuate pathogenic strains of the influenza virus can be solved using recombinant subunit vaccines. The use of various expression systems for rapid production of individual viral proteins in preparative amounts is one of the new approaches to the production of subunit influenza vaccines [21]. 

In one of the popular expression systems, influenza antigens are produced in insect cells using baculoviral vectors carrying the genes of the target antigens. The autographa californica multiple nucleopolyhedrovirus (AcMNPV) is the most commonly used. Sf9 cell lines obtained from *Spodoptera frugiperda* ovarian tissue are typically used for work with AcMNPV. This system can be used to produce various antigens of the influenza A virus. Immunization of mice with the recombinant HA of the influenza H5N1 virus obtained in the baculovirus expression system resulted in the induction of a high level of virus-neutralizing antibodies. However, either an adjuvant or prime-boost immunization using an inactivated influenza H5N1 virus or the recombinant adenovirus carrying the HA gene of the influenza virus was required in order to attain any significant antibody level [22]. 

The ion channel-forming protein M2 is considered the most promising candidate for influenza subunit vaccines. M2 is one of the three influenza A virus proteins that are expressed on the virion’s surface; as opposed to HA and NA, this protein is highly conserved. In viruses circulating in the human population, the M2 protein ectodomain (M2e) has undergone virtually no changes since 1933 [23]; hence, the M2e protein is regarded as a candidate for designing broad-spectrum vaccines. Thus, the possibility of using the cucumber mosaic virus to express the M2e protein of the A H5N1 influenza virus in plants has been demonstrated [24]. 

The low immunogenicity and, therefore, the need for repeated vaccination and use of adjuvants are the drawbacks of recombinant subunit vaccines, as well as of conventional subunit vaccines. One of the ways of solving this problem consists in including molecular adjuvants (ligands of various receptors of the innate immunity system) in the composition of subunit vaccines. The recombinant protein STF2.4×M2e, which is produced in *Escherichia coli * cells and includes flagellin (the toll-like receptor 5 (TLR-5) ligand), has protected immunized mice against a lethal dose of the influenza virus [25]. The safety and efficacy of a vaccine based on this construct was demonstrated in adult volunteers [26]. 

Intramuscular immunization of mice with the recombinant fusion protein 4×M2e *·* HSP70c produced in *E. coli * and consisting of sequential repeats of the M2e and HSP70 proteins of *Mycobacterium tuberculosis* resulted in a significant decrease in weight loss, a reduced viral titer in the lungs, and a less pronounced manifestation of the symptoms of the disease after the mice had been infected with a lethal dose of the influenza A H1N1, H3N2, or H9N2 viruses [27]. 

**Virus-like particles (VLP) **

Virus-like particles(VLP) are antigenic determinants of virions without genomic RNA fragments. Due to the absence of genetic material, VLP are incapable of infecting human and animal cells, which makes them safe [18]. The surface proteins of influenza VLP can be the conformational epitopes of the cells of the immune system as native virions. 

It has been demonstrated in a number of studies that participation of the internal protein of the influenza virus M1 plays the key role in the formation of influenza VLP. This protein is bound to the lipid site of the apical plasma membrane domain, interacts with the surface glycoproteins of the influenza virus, and initiates assembly and budding of VLP containing the lipid membrane of the host cell, with three transmembrane proteins of the influenza virus incorporated into it [28]. 

Influenza VLP have been obtained in various expression systems. Either simultaneous expression of NA or addition of exogenous NA is required to provide efficient release of influenza VLP containing HA from mammalian cells. This fact can be attributed to the ability of active NA to cleave the sialic acids on the surface of the cell membrane [29]. Influenza VLP containing HA can be produced in insect cells even in the absence of NA expression, since the sialic acids in these cells are not bound to N-glycans during post-translational modification [30]. 

**Fig. 2:**
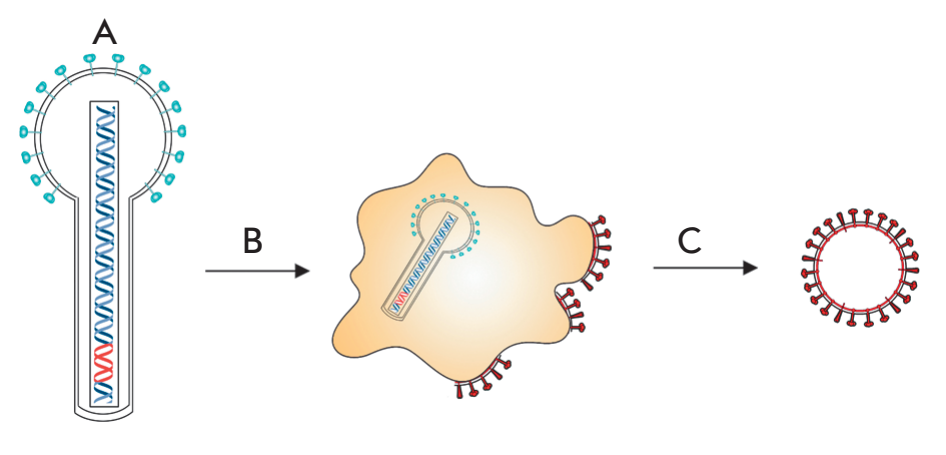
Production of virus-like particles in the baculovirus expression system A – contraction of recombinant baculovirus expressing the gene of influenza antigen, B – transduction of insect cells, C – budding of the virus-like particles

One of the approaches in producing influenza VLP in insect cells assumes the use of recombinant baculoviruses ( *Fig. 2* ) [1]. It has been demonstrated on animal models that the influenza surface antigens within VLP, which have been obtained using recombinant bacoloviruses, induce the production of both antihemagglutinating and virus-neutralizing antibodies and of the effectors of the cellular immune response. Furthermore, the influenza VLP vaccine induces protective immunity against homologous and heterologous strains of the influenza A virus [31]. 

**Fig. 3:**
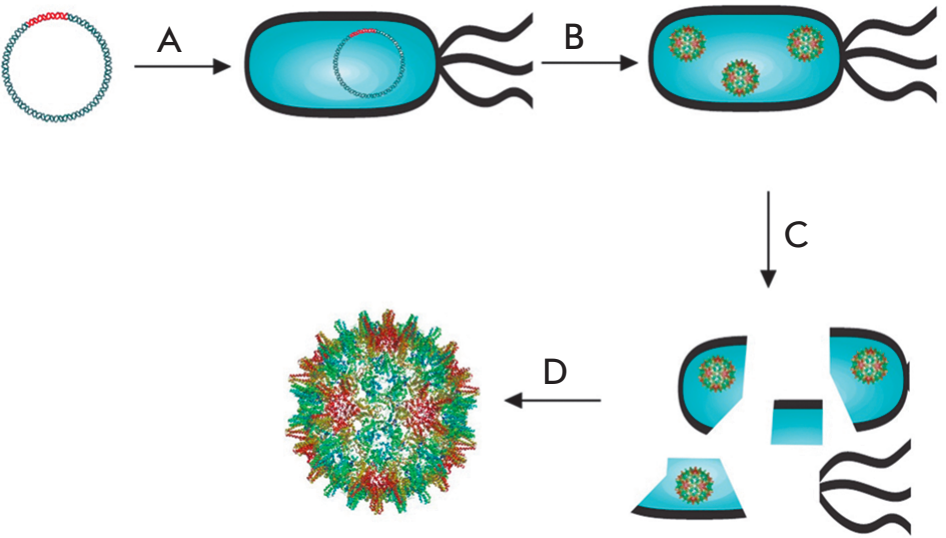
Production of proteasomes. A – transformation of a producer strain by plasmid with the target gene, B – assembly and expression of proteasomes in cells, C – separation of proteasomes from the producer cells, D – purification and production of proteasomes

A vaccine based on VLP carrying antigens of the pandemic influenza A H1N1(2009) virus has undergone phase II clinical trials in 4,563 healthy adult volunteers and has demonstrated that it’s safe and has immunogenicity [32]. 

The use of recombinant baculoviruses for the expression of influenza virus proteins in insect cells results in the accumulation of baculoviruses, along with VLP, in the culture fluid. Since these structures are of similar sizes, it is difficult to isolate VLP from baculovirus particles. Influenza VLP can be generated in mammalian cells using other DNA- and viral vectors. Thus, a system for producing influenza VLP in Vero cells using DNA vectors carrying the genes of the HA, NA, M1 and M2 proteins of the influenza virus has been designed. The use of the modified vaccinia virus Ankara to generate VLP containing proteins of the influenza H5N1 (HA, NA, M1) virus in mammalian cells has been described. These VLP are capable of inducing a protective immune response in mice [33]. 

Thus, production of VLP is a promising direction in the efforts to design new types of influenza vaccines. In order to enhance the immunogenicity, attempts at introducing immune-stimulating components into the structure of influenza VLP have been made. For this purpose, recombinant baculoviruses carrying the flagellin (TLR-5 ligand) gene have been produced. The presence of recombinant flagellin within influenza VLP containing the HA of the influenza A/PR8 (H1N1) virus considerably enhanced the immunogenicity and protective properties of VLP after immunized mice had been infected with the heterologous strain of the influenza virus [34]. 

**Proteasomes **

Nano-sized structures containing the target antigen bound to a carrier consisting of biological macromolecules can be produced using genetic engineering techniques. The so-called proteasomes (a complex of proteins approximately 30-60 nm in diameter, which carries the target antigen on its surface) can be obtained by self-assembly of these macromolecules. Despite the fact that many authors refer to these structures as virus-like particles, opposite to VLP, proteasomes are formed on the basis of a carrier protein. 

Proteasomes are most frequently based on virus coat proteins (e.g., the adenovirus penton [35], human papillomavirus L1 protein [36], hepatitis B virus HBc antigen [37] ( *Fig. 3* ). 

Proteasomes containing the M1 protein of the influenza A virus bound to the structure comprising adenovirus surface proteins (dodecahedron) via the WW domain. The dodecahedron – antigen complex is capable of activating human dendritic cells, which introduce the antigen into cytotoxic T lymphocytes after activation [38]. The human papillomavirus L1 protein [36], the coat protein of Qβ bacteriophage [39], the papaya mosaic virus capsid protein [40], and the woodchuck hepatitis virus antigen have been used as a carrier of the influenza virus M2e protein or various epitopes of the M2 protein. 

The hepatitis B virus HBc antigen, whose monomers can assemble into nano-sized particles, arouses the greatest interest as a carrier protein. These chimeric particles have been used as a carrier protein of the influenza virus M2e protein. The fusion protein M2e-HBc has been produced in *E. coli* cells. Immunization with recombinant М2е-НВc proteasomes has protected mice against a lethal influenza infection even in the presence of preexisting antibodies against the HBc antigen [37]. The system for producing М2е-НВc proteasomes in * Nicotiana benthamiana* cells using the recombinant viral vector based on the potato virus X has been described [42]. 

The ability of proteasomes to carry a large number of antigenic determinants on their surface is an undoubted advantage [36]. However, the immunogenicity of the antigens represented in such a way is not always sufficient. The drawbacks of proteasomes also include their ability to carry small peptides only. 

**Genetic vaccines **

The principle in designing any genetic vaccine consists in that a certain gene or region of the pathogen genome is incorporated into the carrier vector, which is subsequently used for vaccination. These vaccines provide the delivery of genetic material into the host cells and expression of the genes of the pathogen proteins in them. As a result, the pathogen antigens expressed by the cells in the organism are recognized by the immune system, which causes the induction of both the humoral and cell-mediated immune responses. The structure of the target antigens is very similar to that formed upon viral infection. Production of genetic vaccines does not require isolation and purification of antigens and, hence, handling pathogens. Furthermore, the use of various recombinant virus-based vectors can have an additional immunostimulating effect due to the presence of molecular pathogen-associated structures inducing innate immunity in them [43]. 

Among a great variety of genetic vaccines, three major groups can be distinguished: DNA vaccines, bacterial vector-based vaccines, and viral vector-based vaccines. 

**DNA vaccines **

DNA vaccines are bacterial plasmids with the incorporated target gene and regulatory elements providing gene expression after this construct is introduced into the organism [44]. 

The levels of cell-mediated and humoral response induced by the introduction of a DNA vaccine are often insufficient for the development of immunity against pathogens. Therefore, DNA vaccines are typically used along with adjuvants to enhance immunogenicity and together with electroporation and gen-gun procedures (the latter method is delivery using a “gene gun,” a device that injects microscopic DNA-coated particles) to provide better penetration of the genetic material into the cells [10]. 

Phase I clinical trials of the DNA vaccine expressing HA of the avian influenza virus, A/Vietnam/1203/04 (H5N1) where an adjuvant was used, has demonstrated the formation of hemagglutinin-inhibiting antibodies in 47-67% and induction of the T cell response in 75-100% of immunized volunteers. A 3-valent vaccine containing plasmids expressing NP, M2, and HA of the same influenza virus induced the T cell response in 72% of immunized individuals [45]. The use of a DNA vaccine for priming of the immune systems in combination with various other types of vaccines (VLP [46], an attenuated vaccine [47], a recombinant adenovirus [48]) appears rather promising. 

**Recombinant bacterial vector-based vaccines **

Attenuated strains of bacteria, such asBCG, *Listeria* *monocytogenes, Salmonella typhi, S. typhimurium, Shigella flexneri* , etc., are used as bacterial vectors in designing genetic vaccines. Bacterial vectors are characterized by the ability to deliver an antigen to the antigen-presenting cells and the possibility of producing vaccines for intramucosal introduction. The use of bacterial vectors activates the innate immunity as a result of the interaction between the bacterial components and the receptors of the innate immunity system [49]. 

Immunization of mice with *L.monocytogenes* -based bacterial vectors carrying the *NP* gene of the influenza A virus reduced the influenza virus titer in the lungs of infected mice [50]. The safeness and immunogenicity of this vaccine has been demonstrated in volunteers [51]. The use of the *Bordetella pertussis* -based vaccine vector BPZE1 carrying the M2e protein gene of the influenza A virus induced the formation of anti-M2e antibodies in mice and reduced the influenza virus titers in the lungs after the animals had been infected with А/PR8 (H1N1). However, this vaccine failed to provide complete protection when the animals had been infected with a lethal dose of the virus [52]. 

When using bacterial vectors, the resulting immune response is not always sufficient to provide protection; therefore, additional means to enhance the vaccine’s immunogenicity should be employed. The possibility of transferring the plasmid carrying the transgene to other bacteria is a serious downside in the case of bacterial vectors. What’s more, there is a possibility of insertional mutagenesis [53]. 

**Recombinant viral vector-based vaccines **

Viral vectors are recombinant viruses with the target gene and a combination of regulatory elements incorporated into their genome. Viral vectors hold a special position among the existing antigen delivery systems due to the fact that they possess the following properties: a natural mechanism of interaction with cells and penetration into them; they deliver foreign genetic material to the cell nuclei; are capable of providing long-term antigen expression; and their capsid protects the antigen-encoding genetic material [54]. 

Viral vector-based vaccines efficiently activate cytotoxic T lymphocytes, which play a particularly significant role when performing vaccination against intracellular pathogens. These vaccines can have a broad range of activities due to the induction of the T cell response to conserved epitopes that are potentially capable of ensuring protection against various pathogenic strains (including the influenza virus) [55]. 

Viral vectors are capable of activating the innate immunity by binding the genetic material or their capsid proteins to pattern-recognition receptors (TLR, RIG-1, etc.) [56]. Viral vectors are recognized by TLR, such as TLR2, TLR3, TLR4, TLR7, TLR8, and TLR9. The interaction between these receptors and ligands results in the activation of various transcription factors, which leads to the formation of an inflammation locus and rapid activation of the defense reactions of the organism [57]. 

One needs to be guided by the following criteria when choosing a viral vector for genetic immunization: the vaccine should not cause any symptoms of the disease; it needs to be safe for immune-deprived individuals, as well as for elderly people and children; the intrinsic proteins of the recombinant virus should not cause a strong immune response; the viral vector needs to be simple for genetic manipulations and be capable of incorporating large fragments of foreign DNA; the resulting vectors need to have a high viral titer and provide a high expression level of the target antigens; the DNA of a viral vector should not be integrated into the host cell genome after the immunization; and the vector needs to be completely eliminated from the organism after the immune response is induced. Furthermore, the presence of a preexisting immune response to the proteins of the viral vector in immunized individuals is undesirable, since it can considerably reduce the level of the immune response to the target antigen [58]. 

Not all the viruses possess the properties required for the construction of efficient vectors. Poxviruses [59], the recombinant Newcastle disease virus [60], and adenoviruses [61] are those most frequently used today to design viral vector-based influenza vaccines. 

**Recombinant poxviruses **

Poxviruses (Poxviridae) are DNA-containing viruses with a large genome. The vaccinia virus is a poxvirus that is most commonly used as a viral vector; its advantages include simple and inexpensive production, as well as high packaging capacity (up to 25 thousand nucleotide pairs) [59]. Attenuated vaccinia viruses (such as the modified vaccinia Ankara virus and the attenuated NYVAC strain based on the Copenhagen strain) are used for vaccine production. MVA was attenuated by repeated passivation in chicken embryo fibroblasts, which resulted in the loss of a number of genes that are not essential for replication in avian cells and in reduced reproduction in human cells. Attenuation of the NYVAC strain was achieved via deletion of 18 genes; as a result, the virus became replicatively defective for human cells [62]. 

It has been demonstrated that immunization of mice with MVA expressing the HA genes of the highly pathogenic avian influenza H5N1 virus protects mice against both the homologous and heterologous strains of the influenza H5N1 virus, as well as induces virus-neutralizing antibodies and HA-specific CD4 ^+^ - and CD8 ^+^ T cells [63]. The MVA-based vaccine expressing the HA gene of the influenza A/California/07/09 (H1N1) virus proved efficient in the double immunization of mice, macaques, and polecats [64]. The efficiency of the vaccine based on the NYVAC strain expressing the HA gene of the avian influenza A(H5N1) virus was demonstrated for pigs [65]. 

A serious drawback of vaccinia virus-based vectors is the preexisting immunity to this virus, which formed in the human population as a result of immunization against smallpox. Therefore, it is reasonable to use vectors based on the canarypox and fowlpox viruses, against which there are no preexisting antibodies in the human population. Immunization of chickens and ducks with the recombinant fowlpox virus with the HA gene of the avian influenza A virus incorporated into its genome has protected birds against infection with lethal doses of homologous influenza viruses [66]. The high packaging capacity of poxviruses allows one to simultaneously introduce several transgenes (e.g., the HA and NP genes of the influenza A virus) into the genome [66]. However, the canarypox and fowlpox viruses induce a weaker immune response to the target antigens, as compared to that induced by the vaccinia virus, and require repeated immunization or the use of adjuvants [66]. 

**Recombinant Newcastle disease virus **

The Newcastle disease virus (NDV) belongs to the Paramyxoviridae family. This virus has a nonsegmented single-stranded RNA genome containing six genes that encode seven proteins: the NP, Р and V proteins, the M protein, the fusion protein or F protein, HА–NA, and the large polymerase protein L. Since the expression level of each viral protein decreases in the direction from the 3’ to the 5’ terminus of the genome, when NDV is used as a vector, the expression level of a foreign gene can be controlled based on its position in the viral genome. The virulence level and tropicity of NDV depend on the site of the fragmentation of the F protein by nucleases, which is required to provide fusion of the viral coat and the cell membrane. Thus, the virus virulence can be altered via amino acid replacements in the F protein, which can be regarded as a convenient basis for constructing vaccine vectors [60]. 

**Fig. 4:**
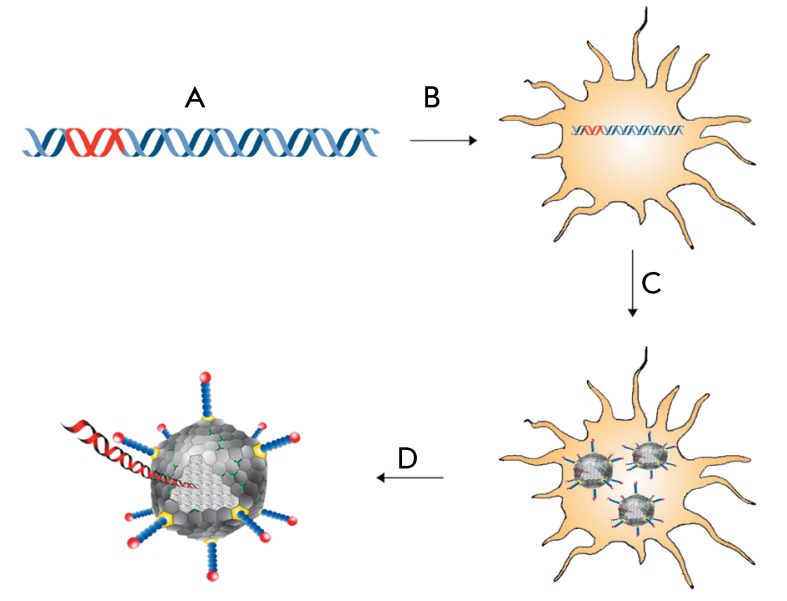
Production of recombinant adenovirus. A – production of genomic DNA of human recombinant adenovirus expressing the genes of influenza antigens, B – transfection of permissive cell line with recombinant virus DNA, C – expression of virus genes in eukaryotic cells and assembly of recombinant virus particles, D – purification of adenovirus virions from a cell suspension

NDV expressing the HA gene of the influenza A/WSN/3 (H1N1) virus has been constructed using the reverse genetics method. Mice has been successfully protected against infection with the influenza A/WSN/3 (H1N1) virus using this construct [60]. NDV expressing the HA genes of the highly pathogenic avian influenza A H5 and H7 viruses has protected immunized birds against infection with lethal doses of homologous influenza A viruses. The efficiency of immunization with the recombinant NDV expressing the HA gene of the highly pathogenic avian influenza A H5 virus was demonstrated in mice [67]. 

In nature, only birds are infected by NDV; hence, humans have no antibodies against this virus. Therefore, there is no problem of preexisting immune response for this viral vector. However, a significant drawback of the vaccine vector NDV is that the consequences of the introduction of recombinant NDV have not been sufficiently studied and it remains unclear whether NDV-based influenza vaccines are safe for humans. Furthermore, NDV is characterized by a low packaging capacity and a complex procedure in constructing vectors carrying several target antigens. Preparative amounts of NDV are produced in chicken embryos, a method which has a number of drawbacks, as shown above [68]. 

**Recombinant adenoviruses **

Recombinant adenoviruses (Adenoviridae) are the best studied and most frequently used recombinant viral vectors. Adenovirus virions consist of a double-stranded DNA molecule surrounded by a protein capsid. 

A number of adenovirus types have been thoroughly characterized (at the genetic level as well). The genomes of most of them have been fully sequenced. Detailed data on the structure, physicochemical, and biological properties of adenoviruses enable their use in designing recombinant vaccines and gene therapeutic agents [61]. Approximately 24% of the genetic vaccines that are currently undergoing clinical trials are vaccines based on recombinant adenoviruses (clinicaltrials.gov) ( *Fig. 4* ). 

Adenoviruses possess significant properties for vaccine vectors: they are capable of providing high levels of expression of the target transgene in the target cell and of transducing both dividing and postmitotic cells. Adenovirus DNA remains in the extrachromosomal form. Adenoviruses can be accumulated to high titers in cell culture. The process of designing a new recombinant adenovirus takes several weeks, which allows a prompt response to a changing epidemiological situation [61]. 

Vaccines based on recombinant adenoviruses against a number of pathogens causing such diseases as malaria, tuberculosis, brucellosis, etc. [69, 70] and various viruses (influenza A virus, human immunodeficiency virus, human papillomavirus, rabies virus, Ebola virus, etc. [71–74]) are currently under development. 

The best studied representative of adenoviruses, human adenovirus serotype 5 (Ad5), is the most commonly used among the adenoviruses used to construct recombinant viral vectors [75, 76]. Replication-defective Ad5 are used to produce vaccines and gene therapeutic agents. In these Ad5, various genome regions (Е1, Е2, Е3, Е4) essential for virus replication are deleted. Cell lines complementing the functions of the removed regions *in trans* have been designed to produce and accumulate these viruses. The vectors enable inserting up to 10,000 bp [77]. 

When injected into the organism, adenoviruses are capable of activating TLR-9 and RIG-1 receptors. The innate immunity is simultaneously activated as a result of adenovirus penetration of antigen-presenting cells [78]. 

Adenovirus-transduced dendritic cells expressing the target antigen or the activated dendritic cells that have captured the antigen produced by epithelial cells act as an interlink between the innate and adaptive immunity systems. Upon mucosal immunization, priming of dendritic cells occurs in mucosal tissues; hence, activated T and B lymphocytes (as well as the memory cells originating from them) acquire the ability to express α4β7 integrin. This molecule allows T and B lymphocytes to migrate through the endothelium layer to submucous tissues to a spot where it is possible to enter in contact with a pathogen [79]. 

It has been demonstrated in experiments with laboratory animals that cross-immunity is developed after mucosal immunization with vaccines of various types [80]. The major component of the adaptive immunity of mucosal tunics is antibodies, which mostly refer to secretory immunoglobulin A (sIgA), to secretory immunoglobulin M (sIgM) to a smaller extent, and to IgG of both plasmatic and local origin. Expression of sIgA presumably determines the cross-protectivity of the vaccine [81]. The other advantages of mucosal vaccines over injective ones include the absence of skin damage during the immunization and lower reactogenicity [82]. 

Thanks to the activation of the innate immune response, intranasal introduction of recombinant Ad5 carrying no transgenes into mice can also protect against the influenza A virus, since it induces production of a broad range of anti-inflammatory cytokines and chemokines (including type I interferons) and nitrogen oxide activates natural killer (NK) cells. The protective effects caused by the introduction of Ad5 carrying no transgenes are retained for at least 3 weeks after a single intranasal immunization. The introduction of recombinant adenovirus protects nonspecifically against low doses of the influenza virus during the period of time that is required for the formation of the adaptive immune response to the target antigens. Thus, the protective effect of Ad5-based vaccines starts almost immediately after the immunization [83]; due to the specific immune answer to the transgene, the effect lasts for over 6 months. 

Vaccines based on recombinant adenoviruses against various influenza A serotypes are currently under development in different countries. One such vaccine has successfully passed phase I clinical trials in the USA and proved to be safe for humans and highly immunogenic for the influenza A H5N2 virus [84]. 

The problem related to the design of influenza vaccines triggering the heterosubtypic immunity that can protect against various strands of the influenza virus is urgent; new approaches in solving the problem have appeared recently. The conformational epitopes of HA have been identified for various influenza A subtypes; broad-spectrum antibodies against these epitopes can be secreted both after the infection and after live virus vaccination [85]. Vaccination with recombinant adenoviral vectors imitates an infection of mucosal cells of the upper air passages, thus providing expression of antigens with a native tertiary structure, which allows to trigger the formation of these cross-reactive antibodies. Recombinant adenoviral vaccines can also induce a strong T cell immune response characterized by a broader spectrum of action compared to that of the humoral immune response. 

The possibility of using recombinant Ad5 to induce a heterosubtypical immune response against the influenza A virus has been studied at the Molecular Biotechnology Laboratory of the Gamaleya Research Institute of Epidemiology and Microbiology. It has been demonstrated that twice-repeated intranasal immunization of mice with the recombinant adenovirus carrying the HA gene of the influenza A H5N2 virus provides high-level induction of specific antibodies against this virus and ensures complete protection of mice against infection with a lethal dose of the H5N2 virus (50 LD _50_ ) [86]. The mice immunized with this recombinant adenovirus were also protected against infection with the influenza H1N1 and H2N3 viruses, which belong to the H1 group (H1, H2, H5, H6, H11, H13, and H16); however, they were not protected against infection with the influenza H3N2 virus, which belongs to the H3 group (Н3, Н14, and Н4) [87, 80]. 

The data obtained allow to assume that a panel of adenoviral vectors carrying the HA genes of influenza A viruses belonging to different groups can be used to design a vaccine that would protect against most epidemic strains of the influenza A virus. 

A serious drawback of using Ad5 as a vector for designing vaccines is that most people have anti-Ad5 antibodies. The presence of these antibodies can significantly reduce immunization efficacy. However, it has been demonstrated in a number of studies that Ad5-based vaccines can avoid the effect of the preexisting immune response upon intranasal immunization (as opposed to parenteral introduction) [88–90]. Upon intranasal introduction of Ad5, the transgene is efficiently delivered through the mucosal barrier. Even a single intranasal administration of Ad5-based vaccines results in prolonged *ex vivo * expression of the transgene despite the preexisting immunity both in laboratory animals and in primates [89]. 

A single intranasal immunization of mice with a recombinant adenoviral vector carrying the HA gene of the avian influenza A H5N2 virus has protected immunized animals against infection with this virus. No differences between the levels of protection were observed in mice with virus-neutralizing anti-Ad5 antibodies present in their blood and in Ad5-naive mice [80]. 

The intranasal immunization of mice with a preexisting immune response to Ad4, recombinant Ad4 carrying the HA gene of the influenza virus, resulted in a lower level of production of antibodies against the influenza virus as compared to that in nonprimed mice and a decrease in the cell-mediated immune response by over 2 orders of magnitude (depending on the dose of recombinant Ad4). However, despite the preexisting immunity, the animals remained fully protected against infection with a lethal dose of the influenza virus [74]. Analogous data were obtained for Ad5 carrying the HA and NP genes of the influenza virus. An increase in the dose of the recombinant adenovirus leveled the reduction of the immune response [90]. 

Incorporation of elements that allow an optimization of the expression level of the transgene into the vector and selection of the optimal dose of Ad5 made it possible to achieve a significant level of transgene-specific CD8 ^+^ cells in immunized animals even at high levels of Ad5-neutralizing preexisting antibodies [91]. 

Thus, recombinant Ad5 carrying genes of various antigens of the influenza A virus are rather promising as candidates for influenza vaccines. They are safe, efficacious, and can be used to design a universal intranasal influenza vaccine. 

## CONCLUSIONS 

The data presented in this review attest to a vigorous research effort aimed at constructing influenza vaccines using new approaches that employ the promise of reverse genetics methods and recombinant technologies, as well as the production of VLP, proteasomes, and subunit vaccines in various expression systems. The new approaches have enabled to achieve significant progress in the design of new influenza vaccines. Some of these vaccines are currently undergoing either preclinical or clinical trials. Among the vectors used to design genetic vaccines, adenoviral vectors hold a special position. They are capable of efficiently penetrating the respiratory mucosal tunic, which makes it possible to achieve mucosal immunization, thus ensuring a lasting presence of the antigen in the organism and activation of the innate immunity. Human recombinant adenoviruses serotype 5 carrying the genes of various antigens of the influenza A virus have the potential of being used as influenza vaccines. They are safe, efficacious, and could allow to design a universal influenza vaccine delivered intranasally. Upon immunization, the recombinant adenovirus acts as an adjuvant; it is capable of boosting immunity with respect to the transgene. Producing this vaccine takes several weeks, which would allow to respond promptly to a changing epidemiological situation. Recombinant adenoviral vectors carrying the HA genes of various subtypes of influenza A viruses can be used to form a heterosubtypic immune response against most epidemic variants of the influenza A virus. Thus, adenoviruses can be used to design a universal recombinant influenza vaccine. 

## Abbreviations

WHO: World Health Organization
DNA: deoxyribonucleic acid
HA: hemagglutinin
NA: neuraminidase
VLP: virus-like particles
RNA: ribonucleic acid
NP: nucleoprotein
DC: dendritic cell
APC: antigen-presenting cell
Ad: adenovirus
NK: natural killers
